# Supplementary material for: A Type Ib Crustin from Deep-Sea Shrimp Possesses Antimicrobial and Immunomodulatory Activity
Source: Int J Mol Sci. 2022 Jun 9;23(12):6444. doi: 10.3390/ijms23126444 (PMC9223358; doi:10.3390/ijms23126444)

Supplementary Data

Table S1. The primers used in this study.

Primer name	Sequence (5'-3')
F1	5'- GGACACCAACAACGTGACTGC -3'
R1	5'- CCGAGAAGGCTTGCAGGTATG -3'
F2	5'- GAACCTCGAAATCTTGCTACGACACCTGC -3'
R2	5'- GCAGGTGTCGTAGCAAGATTTCGAGGTTC -3'
F3	5'- GACCACCATACCTCCAAGCCTTCTCGG -3'
R3	5'- CCGAGAAGGCTTGGAGGTATGGTGGTC -3'

Figure S1. SDS-PAGE analysis of Crus2 and Crus2DC. Purified Crus2 (A) and Crus2DC (B) were analyzed by SDS-PAGE and viewed after staining with Coomassie brilliant blue R-250. M, molecular marker.

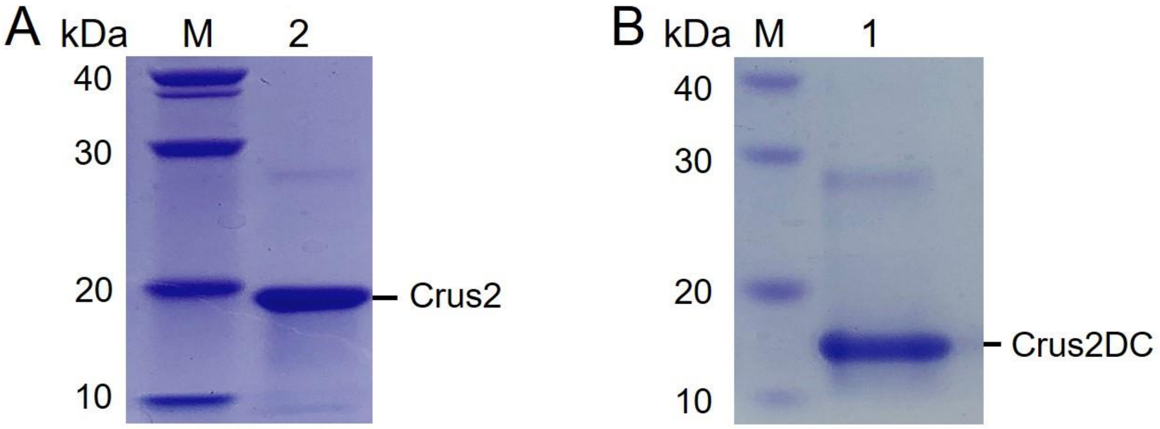

Supplement: Supplementary file 1 [file ijms-23-06444-s001.zip › ijms-1762192-SI.pdf]
